# Predicting Fatigue and Psychophysiological Test Performance from Speech for Safety-Critical Environments

**DOI:** 10.3389/fbioe.2015.00124

**Published:** 2015-08-25

**Authors:** Khan Richard Baykaner, Mark Huckvale, Iya Whiteley, Svetlana Andreeva, Oleg Ryumin

**Affiliations:** ^1^Speech Hearing and Phonetic Sciences, Psychology and Language Sciences, University College London, London, UK; ^2^Mullard Space Science Laboratory, Centre for Space Medicine, University College London, Dorking, UK; ^3^Gagarin Cosmonaut Training Centre, Star City, Russia

**Keywords:** fatigue, speech, computational paralinguistics, bioinformatics

## Abstract

Automatic systems for estimating operator fatigue have application in safety-critical environments. A system which could estimate level of fatigue from speech would have application in domains where operators engage in regular verbal communication as part of their duties. Previous studies on the prediction of fatigue from speech have been limited because of their reliance on subjective ratings and because they lack comparison to other methods for assessing fatigue. In this paper, we present an analysis of voice recordings and psychophysiological test scores collected from seven aerospace personnel during a training task in which they remained awake for 60 h. We show that voice features and test scores are affected by both the total time spent awake and the time position within each subject’s circadian cycle. However, we show that time spent awake and time-of-day information are poor predictors of the test results, while voice features can give good predictions of the psychophysiological test scores and sleep latency. Mean absolute errors of prediction are possible within about 17.5% for sleep latency and 5–12% for test scores. We discuss the implications for the use of voice as a means to monitor the effects of fatigue on cognitive performance in practical applications.

## Introduction

There are a variety of safety-critical environments for which operator fatigue is a significant risk factor, including transport, mining, and aeronautics. In response to this risk, a variety of methods have been developed to estimate the fatigue level of an operator. Some of these are accurate but based on obtaining physiological measurements, which require expensive or intrusive equipment. Since in some safety-critical environments, operators are engaged in regular verbal communication, a fatigue estimation method based on the analysis of speech might provide a cheaper and less intrusive alternative.

To develop a reliable means of predicting fatigue from speech requires an objective measure of level of fatigue for use in the training of models. However, previous studies that have explored the prediction of fatigue from speech have relied on subjective ratings of sleepiness as the outcome parameter, and these ratings do not always correlate well with objective measures obtained from behavioral tasks.

In this paper, we describe an experiment in which a group of subjects were kept awake over a 3-day period within which regular measurements were made both of their speech and of their performance on a standard set of psychophysiological tests (PPTs). We investigate the relationship between time kept awake (“sleep latency”) and time-of-day (“phase”) with their test performance. We show that test scores are poorly predicted from sleep latency and phase in these data. We show that a model based on speech features, on the other hand, can improve prediction of the test scores, and in addition, make good predictions of sleep latency.

The paper is organized as follows. Section “Background” gives a background to the topic, outlining some existing literature on the effects, measurement, and prediction of fatigue in safety-critical environments. Section “Corpus Collection and Cleaning” describes the methodology for acquiring the corpus of speech and performance measures. Section “Voice Features” describes the voice features calculated from the speech recordings. Section “Analysis of Psychophysiological Tests” gives an analysis of how the PPT scores change over time. Section “Model Training” describes the process by which predictive models of fatigue and of test scores are built from speech features and presents an evaluation of their performance. Finally, Section “Summary and Conclusion” summarizes the key findings and discusses some issues of practical implementation.

## Background

In safety-critical environments, fatigue is a significant risk factor. One of the clearest examples of this is in transportation, where driver fatigue is widely considered to be an important contributory factor in fatal and serious accidents (Horne and Reyner, 1995; Lyznicki et al., 1998; Pierce, 1999; Philip et al., 2001; Dobbie, 2002; Armstrong et al., 2011; FMCSA, 2011). It is difficult to pinpoint the exact proportion of accidents caused by fatigue but the consensus of scientists studying safety and accident prevention is that fatigue is the largest identifiable and preventable cause of accidents in transportation, accounting for around 15–20% of all accidents (Åkerstedt, 2000). Fatigue is just as significant a risk in other safety-critical settings where vigilance is important, such as aviation (Rosekind et al., 1994) and space flight.

It is important to note that “fatigue” has been used to describe a variety of phenomena relating to degradations of performance due to physical or mental exertion, lack of sleep, extreme tiredness, etc. A review of definitions is given in (Philips, 2015), and it is important to be clear about the relevant aspects of fatigue under consideration. In this work, the most relevant aspect of fatigue considered is that of sleepiness/wakefulness.

Sleep deprivation studies have shown that fatigue tends to impair cognitive and motor performance (Pilcher and Huffcutt, 1996), leading to reduced reaction times (RTs), vigilance, and memory (Horne and Pettitt, 1995; Lorenzo et al., 1995; Maquet, 2001; Stickgold, 2005). The major causes of fatigue are phase, sleep latency, duration of sleep, duration of work, task-specific factors, and any relevant sleep pathologies (Åkerstedt, 2000; Williamson et al., 2011). See Williamson et al. (2011) for a review of the evidence behind these identified causes of fatigue. Sleep latency is a particularly important factor in fatigue-related accidents; an analysis by the Federal Motor Carrier Safety Administration (FMCSA) of fatigue-related accidents suggested a logistic relationship between sleep latency and proportion of crashes (FMCSA, 2011).

Fatigue can be measured and quantified in a variety of ways. The subjective measures are the simplest of these. Some of the commonly used measures are the Epworth Sleepiness Scale (ESS) (Johns, 1991) and the Karolinska Sleepiness Scale (KSS) (Åkerstedt and Gillberg, 1990). The KSS is commonly used, in part, due to its simplicity and the speed with which it can be administered (a simple 9 point scale with annotated anchors), whereas the ESS, which asks subjects to rate from 0 to 3 a number of situations for likelihood of dozing, tends to be used more in clinical settings since it is based on general information about a subject’s lifestyle.

Gillberg et al. (1994) showed that for subjects kept awake overnight, KSS scores correlated with performance on a visual vigilance task and a RT task with *r* = −0.62 and 0.71, respectively. This indicates that around 38–50% of the variation in performance was explained by subjective ratings.

There are some problems, however, with using self-reported measures of sleepiness. First, subjects will be biased in various ways and as a result there is considerable variability in individual abilities to recognize fatigue (Horne and Baulk, 2004; Kaplan et al., 2007). In studies validating the KSS, correlations between RT tests and KSS scores have been moderately strong, with *r* = 0.57 (with a SD of 0.25 across subjects) in Kaida et al. (2006), and between 0.49 and 0.71 for individual subjects in Gillberg et al. (1994). Yet, a large study involving shift workers at a paper mill carried out by Åhsberg et al. (2000) showed no correlation between the results of RT tests and KSS scores. Thus, it seems likely that confounding variables and individual differences between subjects have a very large effect upon the relationship between subjective measures of sleepiness and performance. Similarly, Williamson et al. (2011) point out that in many studies involving shift work, subjective ratings of sleepiness are typically higher in the morning and lower in the afternoon, even though risk of incidents tends to show the reverse trend; it is further noted that one possible explanation for this could be that risk is substantially more affected by factors, such as time awake than by subjective ratings of sleepiness.

Instead of subjective reports on sleepiness, a wide variety of objective measures can also be used to quantify fatigue. One type of objective measurement of fatigue is to use a performance measurement as a proximate measure. Psychological or physiological tests of this type include RT, vigilance, or cognitive tests (Jackson and van Donger, 2011).

The estimation of fatigue level from subjective reports or from the results of PPTs can be informative in the scientific study of sleepiness and sleep pathologies. In safety-critical environments, however, where it would be helpful to monitor or predict fatigue to reduce risk, these approaches are impracticable. Due to this, there has been increasing interest over the last few decades in generating models to predict fatigue based on objective measurements. One such type of this model is known as a biomathematical model, which makes predictions from information of the known major factors of fatigue, such as phase, sleep latency, and duration of sleep. Many such biomathematical models exist (Mallis et al., 2004), and some have been developed for specific applications, such as aerospace (Mallis and Mejdal, 2003). In general, these models are used to aid with fatigue risk management for transport and aviation organizations, but are not relied upon in isolation. Part of the reason for this is that due to the types of input to the model, the models are unable to account for individual differences in subjects (other than those which manifest directly in duration of sleep), and so the prediction accuracy for any individual suffers even as the general trends are predicted correctly. For a comparison of models see van Dongen (2004).

Another class of models being developed to predict fatigue is computer-vision-based monitoring systems. A review of these can be found in Barr et al. (2005). These models focus on aspects of the face, which give cues to fatigue, examples of which include blink rate and the proportion of time the eyelids cover more than 80% of the eye. Sometimes, these models also include other non-vision-based inputs, measuring aspects of physiology, such as heart rate. Recent models of this kind have accuracy ratings of 90% for predicting behavioral outcomes, such as simulated driver crashes (Vural et al., 2008).

Even more accurate ways of monitoring fatigue are possible using more intrusive physiological measurements. Begum (2013) reviews the capacity of electroencephalography (EEG), electrocardiography (ECG), elektrookulogram (EOG), and pulse oximetry measurements to describe fatigue.

While the vision-based physiological approaches may be accurate, measuring these features presents a significant challenge to user acceptance in many practical applications because of the additional expensive or intrusive equipment required. By contrast, a cheap and non-intrusive fatigue monitoring system could be implemented if it were possible to predict fatigue from voice. This would be particularly useful in those situations requiring drivers or operators to regularly communicate by speaking, (e.g., in aviation, aeronautical, or mining transportation industries).

Existing research has identified a number of vocal correlates of fatigue. Vogel et al. (2010) demonstrated that when subjects were kept awake for a period of 24 h, the durations of their pauses gradually increased for read speech, and variation in the fourth formant frequency decreased for sustained vowel sounds. The study concluded that speech analysis provides objective data on central nervous system functioning and therefore on fatigue.

Krajewski et al. (2009) used machine learning methods to generate models for predicting fatigue from read speech samples of formulaic operator system communication. The test corpus was based on subjects giving ratings on the KSS indicating how fatigued they felt. The most accurate model was able to recognize and distinguish between samples classified as “slightly fatigued” and samples classified as “strongly fatigued” with a recognition rate of 83.8%. A variety of similar models were generated for the Interspeech 2011 speaker state challenge (Schuller et al., 2011), with similar degrees of success. Another version of this type of model has been developed with to predict “microsleeps” with similar success (Krajewski et al., 2008). Other variations also exist, such as the model developed by Günsel et al. (2013), which uses prosodic features extracted by psychoacoustic masking, and the model developed by Thakare (2014), which utilizes automatic speech recognition (ASR) to identify key phonemes from which to extract features. This latter approach would require a robust ASR system in operational environments to account for environmental noise, reverberation, and channel distortions.

In summary, these studies of fatigue prediction from speech shows that features can be computed from the speech signal to categorize speakers as fatigued/not-fatigued with modest performance based on a subjective rating of sleepiness. However, these studies still fall short for application within safety-critical environments, since (i) subjective ratings are not always reliable, (ii) system performance has not been compared to predictions based on time alone, and (iii) fatigue should be considered to be a continuous rather than a categorical measure.

In this work, we address some of the weaknesses of previous studies. We describe a data set comprising speech recordings and objective psychophysiological test results collected from speakers over an extended period of wakefulness. We build predictive models of fatigue from both speech and time using regression methods that treat fatigue as a continuous measure. This allows us to estimate the additional value of speech information over time alone for objective measures of fatigue. Our goal is to test whether the prediction of objective fatigue from speech is a tractable problem and to estimate the possible performance of machine learning approaches.

## Corpus Collection and Cleaning

Seven aeronautical professionals (six male and one female) as part of their training took turns participating in a wakefulness study wherein each remained in an isolation chamber for a period of approximately 60 h. The isolation began at 10 a.m. on day 1 and concluded around 9 p.m. on day 3. During the isolation, the subjects did not sleep and among other duties were tasked with carrying out a standard battery of PPTs approximately every 6 h. In addition, the subjects recorded several minutes of read speech at regular intervals of approximately 6 h (although at different times to the PPTs). This section describes these activities, and the process undertaken to construct a data corpus.

### Psychophysiological tests

The full battery of PPTs consisted of 12 tasks. From these five particular sub-tests measuring RT, memory, and cognition were selected for further analysis as these are known to relate to fatigue (see Background). Table 1 gives a description of these PPTs.

**Table 1:** **Psychophysiological tests selected for analysis**.

S No.	Type	Measures	Description
1	Simple RT	Mean reaction time in milliseconds averaged over 57–60 trials	A monitor displays occasional flashes of light. Subjects must respond to every third flash by pressing a button on a handset as quickly as possible
2	Planned RT	Mean timing error in milliseconds averaged over 50 trials	A monitor displays a colored bar growing in an arc within the border of a circle. After some time, a line appears ahead of the forward end of the bar, and the subject must press a key to stop the bar as close to the line as possible
3	Memory (pictures)	Count of pictures missed and incorrect selections	A monitor displays 16 pictures in a 4 × 4 grid for 20 s. The pictures disappear and are immediately replaced by 64 pictures containing the original 16. The subject has 60 s to identify the original set of pictures
4	Memory (numbers)	Count of numbers missed and incorrect selections	A monitor displays 12 numbers from the range 1–100 displayed in a 3 × 4 grid for 20 s. The numbers disappear for 15 s, and then a 5 × 6 grid of numbers appears containing the original 12 numbers. The subject has 60 s to identify the original set of numbers
5	Cognition	Total time taken to complete task (in seconds)	A monitor displays a 7 × 7 grid containing randomly positioned red and black numbers (for example, 1–25 in red and 1–24 in black). The subject performs three tasks as fast as possible: (i) click the black numbers in ascending order; (ii) click the red numbers in descending order; (iii) alternately click red and black numbers, with the red numbers descending and the black numbers ascending

It was noted that the resolution of scores for the memory tests were fairly small (only 12 or 16 states), and it was decided that instead of using two individual memory performance scores it would be better to combine these data in a way which also increases the overall resolution of the performance measures. This was done by summing the errors for both memory tests (where errors include both failing to select a picture or digit from the original set, and selecting pictures or digits which did not feature in the original set). In this way, four measures of performance are considered: simple RT in milliseconds, planned RT in milliseconds, memory in total number of errors, and cognition in seconds. In all cases, higher values represent poorer performance.

### Speech recordings

In total, 74 recordings of speech were collected (4 subjects produced 10 recordings each, 2 subjects produced 11 recordings, and 1 subject produced 12) at approximately 6 h intervals. The inconsistent number of recordings per subject occurred because a few subjects additionally provided a “baseline” recording the day prior to the experiment (at the same time of day as the experiment begun) and a couple of subjects missed the final recording on the third day. The selected reading materials were excerpts from a novel displayed on a screen, and recordings were made on a Roland R-05 digital recorder sitting on the desk in front of them. Recordings were collected at 24-bit resolution at 44,100 samples per second. Subjects were able to decide themselves how much to read so recording durations varied between 105 and 495 s.

Since the outcome of the modeling work is prediction of level of fatigue, it was necessary to assign each recording a number related to the fatigue level of each subject at the time of recording. There were two possible sources of fatigue level: one computed from the test scores or one computed from sleep latency and phase.

### Aligning speech and PPT data

An alignment process is required for the speech and PPT corpus. Since the PPT and speech data were recorded at different times throughout the experiment it is necessary to use some method of aligning the speech and PPT data for comparison.

A simple method, utilized in this work, is to uniquely assign the nearest PPT result (in time) to each recording; with an average of 3 h difference between the start time of the speech recording and the start time of the PPTs. Other possible methods could be used; for example, PPT scores could be linearly interpolated between adjacent recording times, or a more sophisticated approach could involve modeling the change in PPT scores with time. However, doing this involves making assumptions about the relationship between PPT scores and sleep latency, so it was considered preferable not to use these methods.

## Voice Features

In order to build models describing or predicting fatigue based on speech, it is necessary to quantify descriptive aspects of that speech, these are known as features. Building a large vector of features describing many different aspects of the speech increases the likelihood that some of these features may be related to fatigue. Features were therefore extracted representing variation of the speech recordings in the time, frequency, and modulation domains.

### Feature extraction

The following steps were taken to generate the feature vectors:

1. The speech recording waveform is first pre-emphasized and divided into 50 ms Hamming-windowed sections overlapping by 10 ms.
2. A fast Fourier transform (FFT) is applied to each window and a bank of triangular filters is used to calculate a smoothed spectrum. The filters are 200 mel wide and spaced by 100 mel.
3. A cosine transform of the log-compressed smoothed spectrum is taken to generate 19 mel Frequency Cepstral Coefficients (MFCCs) per frame.
4. The first and second temporal differences of the MFCCs are computed.
5. The autocorrelation of each window is computed and interpolated onto a log delay axis.
6. A cosine transform of the log delay autocorrelation function is taken producing 19 autocorrelation coefficients (ACCs) per frame.
7. The first and second temporal differences of the ACCs are computed.
8. The energy of each window is calculated, and the first and second temporal difference is computed.
9. The distributions of the MFCCs, ACCs, and energies are used to calculate summary statistics. These are based on the 5, 10, 25, 50, 75, 90, and 95% quantiles together with skewness and kurtosis.
10. Finally, the speech recording is band-pass filtered between 300 and 3500 Hz, rectified and low-pass filtered at 80 Hz to generate a temporal envelope trace. The modulation spectrum of the temporal envelope was calculated using 40 band-pass filters logarithmically spaced between 0.1 and 50 Hz.

In total a feature vector containing 1093 parameters was generated for each recording. The MFCCs were chosen to describe the shape of the frequency spectrum of the speech, and the first and second temporal differences to describe the changes in this shape over time. Similarly, the ACCs describe the shape and structure of the short-time speech waveform, while the modulation features describe longer temporal structure. In general, it is preferable to produce a large feature space which captures the changes in characteristics of the voice, which would reasonably be expected, vary with changes in fatigue. Some of these features additionally have precedent in the literature; for example, MFCCs, as well as their time derivatives and second time derivatives, have been shown to be promising for describing fatigue (Greeley et al., 2013). It has also been demonstrated that autocorrelation-based features can also be useful for representing speech (Ando, 2013), although these features have not yet been used in the construction of predictive models in paralinguistics.

It is clear that in generating so many features, some are bound to have little or no bearing upon the fatigue of the speaker. However, it is important to first maximize the descriptive power of the feature set before pruning away those aspects which do not aid in prediction. The former task is known as feature extraction and the latter as feature selection, and both constitute important parts of model construction.

## Analysis of Psychophysiological Tests

**Figure 1:**
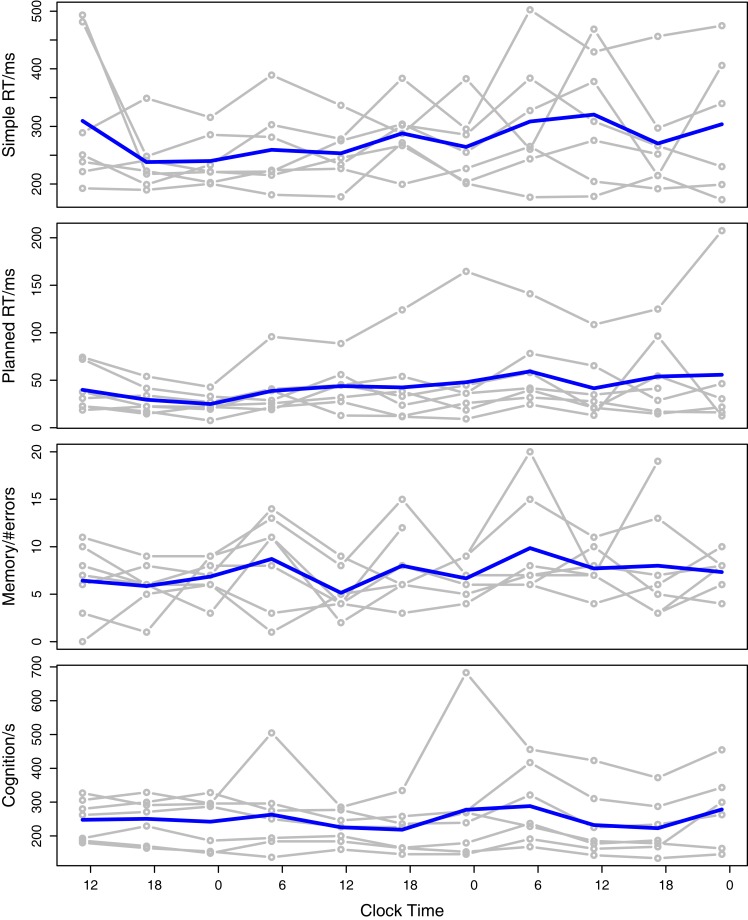
**Psychophysiological test scores plotted against the clock time in the isolation experiment**. The gray lines indicate the subject scores and the blue line is the mean score across subjects.

An analysis of the PPT data and their relation to sleep latency and phase was carried out. Figure 1 shows the PPT scores plotted across the duration of isolation. Each subject’s data is plotted in gray with the overall mean across subjects in blue.

To explore the effects of subject, sleep latency and phase isolation on the PPT scores, a mixed-effects linear regression model was estimated for each test. In this model, the test score for any particular subject at any particular time is predicted from three factors: sleep latency (fixed factor), phase (fixed factor), and a random factor that represents the average test performance of each subject. The phase parameter was derived from the time of day using:

$$P=\cos\left(\omega \left(t-\alpha \right)\right)$$

where *t* is the time of day, α is a constant set to 3 h, and ω is set to 2π*f* such that one cycle spans 1 day (i.e., =1/24). The selection of α = 3 h shifts the positive peak of the cycle to 3 a.m., which is close to the typically quoted time of the lowest point in the circadian rhythm (Duffy et al., 2002). Linear mixed-effects models were trained using the R statistics package “lme4” (Bates et al., 2014). Table 2 provides the regression coefficients and significance values for each fixed factor for each test. The significance values are estimated using ANOVA by building two models, with and without the factor, to see if leaving out the parameter makes a significant reduction in the accuracy of the model.

**Table 2:** **Mixed-effects linear regression model describing the relationship between PPT scores, sleep latency, and phase**.

Test	Sleep latency	p(Sleep latency)	Phase	p(Phase)
Simple RT	0.653	0.107	−3.10	0.774
Planned RT	0.403	0.00347*	1.22	0.734
Memory	0.0286	0.156	0.785	0.149
Cognition	−0.0256	0.939	25.8	0.00471*

The * marker indicates significance at *p* < 0.05.

For the planned RT test sleep latency had a significant effect on the score, and for the cognition test phase had a significant effect on the score. All other effects were non-significant at *p* < 0.05. The effect of phase on the modeling was not sensitive to the choice of the 3 a.m. reference value, and similar outcomes were found for phase references between 1 a.m. and 6 a.m.

Although these results agree with the graphs in Figure 1, they are somewhat surprising, since it has been generally noted that sleep latency and phase are important predictors of fatigue, and that RT, memory, and cognition are impaired by fatigue. We suggest below some origins for the weakness of the relationships found here.

First, it may be that these subjects were motivated to perform fairly well, even with sleep deprivation, so were able to focus their attention for the duration of the tests. Horne and Pettitt (1995) showed that subjects could maintain baseline performance for 36 h on an auditory vigilance task when given a monetary incentive, and continue to perform better than subjects with no incentive beyond this time. Cote et al. (2009) saw subjects able to recruit additional mental effort to combat the effects of fatigue in an electroencephalography study. In this experiment, the subjects were aeronautical professionals in training and may have believed that they were going to be judged on their PPT performance, which gave them an incentive to perform well.

Second, it has been demonstrated that there are significant individual differences in the size of the effect of sleep deprivation (Frey et al., 2004). In Frey et al. (2004) the subjects most resilient to sleep deprivation had similar mean performance scores before and after sleep deprivation, but much greater variation after being deprived of sleep. The subjects in this experiment were aeronautical professionals likely to have been selected for their ability to concentrate on certain cognitive tasks, so may show better than average resilience to sleep deprivation.

Third, since prior to the experiment subjects had only limited exposure to the test battery, it is possible that there was a learning effect. Since any learning effect would result in better performance over repeated practice this may have concealed some of the fatigue effect, resulting in a smaller than expected change in score over time.

Under any of these interpretations, it is clear that to proceed with our analysis of the effect of fatigue on speech we now have two possible approaches. Either we assume that the PPT scores do indeed indicate level of fatigue and aim to predict these from characteristics of the speech or we assume that the sleep latency and phase indicate level of fatigue and aim to predict these from speech. A comparison of our modeling results may shed light on the relative value of these two measures.

## Model Training

With the speech features generated and the initial analysis complete, it remains to generate models to predict level of fatigue from the speech recordings. In the Section “Models Predicting Sleep Latency and Phase from Speech,” we consider models that predict sleep latency and phase from the speech, while in the Section “Models Predicting Psychophysiological Test Performance from Speech,” we consider models that predict the PPT scores from speech.

### Models predicting sleep latency and phase from speech

In this section, models are trained to predict fatigue, on the assumption that latency and phase are the primary components of interest as suggested by Åkerstedt (2000) and Williamson et al. (2011). Predictions of fatigue according to sleep latency on the same data set is presented in Baykaner et al. (2015), where a binary classifier for fatigued/not-fatigued (based on sleep latency) achieved a classification accuracy of 80% for speaker independent features and 90% for speaker-dependent features. In contrast in this work, we train regression models for the continuous prediction of sleep latency and phase.

#### Model Construction

Before constructing the prediction models the 1093 extracted speech features described in the Section “Feature Extraction” were normalized within subjects by gaussianization (Chen and Gopinath, 2001). This process maps each feature value distribution onto a Gaussian distribution. This not only conditions the features values but also makes features more similarly distributed across speakers so that the sensitivity of each feature to a particular voice is minimized. This is important because without some type of subject normalization it is possible to train models which appear to perform well but, in fact, simply predict which speaker is talking.

Predictive models were trained using 100 randomized 10-fold cross-validations. Since the corpus is relatively small it is difficult to reserve sufficient data both for testing and training in isolated sets, so a cross-validation procedure was considered more appropriate, and by carrying this out 100 times with the order of the data randomized each time, the confidence in the reliability of the results can be improved. The selected model training algorithms were multilinear regression (MLR) and support vector regression (SVR) using the “SMOreg function” (Shevade et al., 1999). The SVR method was implemented using a linear kernel with two tested complexity parameters (*C* = 1 and *C* = 0.01). The corpus was not sufficiently large to allow for a separate development set involving rigorous hyper-parameter training (as would be necessary with more complex SVR kernels), so only two cases are tested here to give a broad indication of how the trade-off between model flexibility and robustness affects prediction accuracy.

#### Results

Models were evaluated by comparing metrics calculated as averages and SDs across 100, 10-fold cross-validations. Table 3 shows the relative performance of the models.

**Table 3:** **Cross-validation model training for sleep latency, and phase from speech features**.

Test	Model	Average performance over 100, 10-fold CVs		
		R	MAE	RAE
Sleep latency	Null model	0.00 (0.00)	993.90 (200.82)/min	100 (00.00)%
	MLR	0.71 (0.18)	726.53 (190.27)/min	73.77 (19.85)%
	SVR (*C* = 1)	0.70 (0.19)	756.36 (219.35)/min	77.95 (26.84)%
	SVR (*C* = 0.01)	**0.73 (0.19)**	**629.70 (167.38)/min**	**64.44 (17.69)%**
Phase	Null model	0.00 (0.00)	352.85 (75.21)/min	100 (00.00)%
	MLR	0.43 (0.35)	379.58 (106.43)/min	112.86 (46.52)%
	SVR (*C* = 1)	**0.44 (0.34)**	369.63 (101.17)/min	109.72 (44.03)%
	SVR (*C* = 0.01)	0.30 (0.39)	**321.82 (86.85)/min**	**92.16 (20.17)%**

*Average metrics are displayed with SDs shown in parentheses. Bold shows best performing systems*.

The results are given in terms of correlation (R), mean absolute error (MAE), and relative absolute error (RAE). R indicates the strength of the linear relationship between the predictions and observation. The MAE answers the question “how close is the average estimate to the actual value?” RAE is the ratio of the MAE of the trained models to the MAE of the equivalent null model (i.e., a model that always predicts the mean parameter value). Thus, an RAE score below 100% indicates predictions which have smaller error than a null model, and an RAE of 50% indicates that there was half as much error.

The cross-validation procedure shows that the speech model predictions correlated with sleep latency at around *R* = 0.71–0.73, explaining 50–53% of the variation in the data, whereas phase predictions were poorer and had correlations of *R* = 0.3–0.44, explaining only 9–19% of the variation. The MAEs and RAEs show substantially lower error for the sleep latency predictions than for the null model, whereas for phase the average prediction, errors were similar to the null model. It is important to note that the phase MAEs are artificially high because some errors are calculated as being >12 h whereas the error should be calculated as the smallest difference in time between prediction and observation; for example, a predicted phase of 23:00 for an observed phase of 01:00 will be assigned an error of 22 h, whereas it should be assigned an error of 2 h. Since prediction errors of >12 h are very infrequent; however, the effect of this inaccuracy is small. For example, recalculating a single 10-fold cross-validation with the adjusted MAEs resulted in MAE = 309.60, and MAE = 343.53 for the SVR (*C* = 0.01) and MLR, respectively. Dividing the MAEs by the range of possible observations in each case (i.e., 3600 min for sleep latency and 1440 min for phase) demonstrates that the best average prediction error was equivalent to 17.49% of the range for sleep latency, and 22.35% of the range for phase.

**Figure 2:**
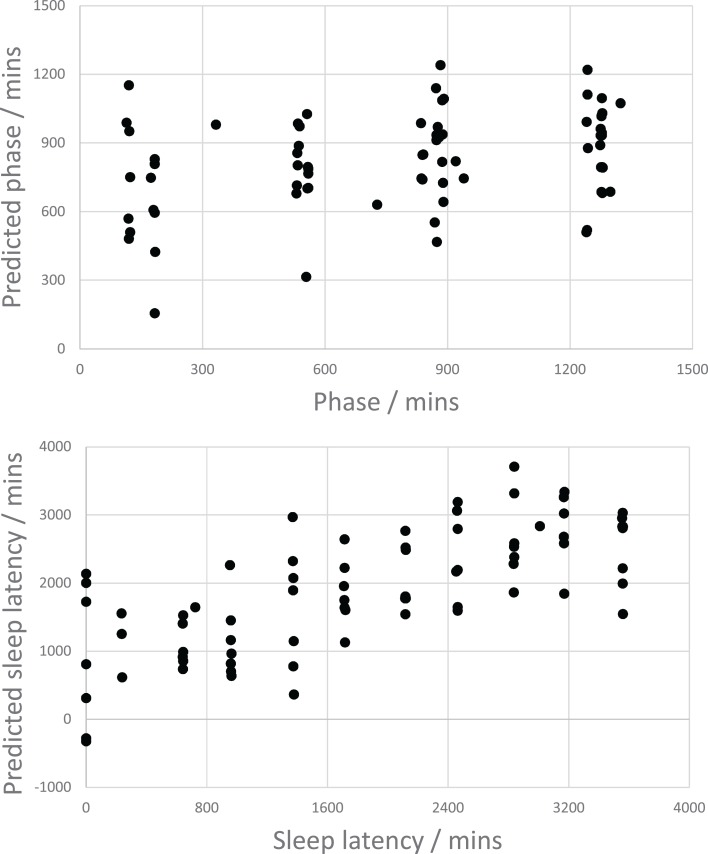
**Scatter plots showing the relationships between speech-based model predictions of phase (top) and sleep latency (bottom)**.

Figure 2 shows the predicted phase and sleep latency from all test folds of a single 10-fold cross-validation of the SVR (*C* = 0.01) model plotted against the respective observed values. Although the phase prediction error was similar to that of the null model, the scatterplot in Figure 2 indicates that the phase model does have a small degree of predictive power for separating the early and late periods of the day. As expected, the scatterplot for sleep latency shows a positive linear correlation, indicating that the sleep latency model performs reasonably well.

### Models predicting psychophysiological test performance from speech

As discussed in the Section “Aligning Speech and PPT Data,” the PPTs and speech recordings were also not temporally coincident. As a result, the PPT scores must be aligned to the speech recordings. This was done by selecting the nearest recording time to each PPT score and assigning the score (uniquely) to that recording. In addition to this alignment, however, this process resulted in six samples being excluded. This is because 5 of the 74 speech recordings were baseline recordings made the day prior to the experiment for which no equivalent PPT score is associated, and a remaining recording was made only a few minutes after the end of one subject’s isolation (leaving no unique PPT scores to assign to it). The corpus thus has 68 aligned samples.

Before constructing models to predict the four sets of PPT scores described in the Section “Psychophysiological Tests,” the PPT scores were *Z*-standardized within subjects to account for individual differences in performance. Without standardizing the PPT scores any constructed models may be trained to predict the subject (that ordinarily has higher or lower scores), rather than the change in subject performance, which is actually of interest.

Models were constructed to predict standardized PPT scores using the same subject feature normalization and model training procedures as described in the Section “Results” using 100, 10-fold cross-validations. Table 4 shows the results of the constructed models together with the performance of a simple linear regression model based on two features of sleep latency and phase (denoted as “Time MLR”) for comparative purposes.

**Table 4:** **Performance results for models constructed to predict psychophysiological test scores from time only or from speech features using SVM and MLR approaches**.

Test	Model	10-Fold performance		
		R	MAE	RAE
Simple RT	Null model	0.00 (0.00)	0.76 (0.21)	100.00 (0.00)%
	Time MLR	0.14 (0.39)	0.75 (0.21)	99.91 (13.21)%
	Speech MLR	**0.49 (0.35)**	**0.64 (0.21)**	**86.57 (26.75)%**
	Speech SVR (*C* = 1)	**0.48 (0.36)**	**0.64 (0.21)**	**87.01 (26.50)%**
	Speech SVR (*C* = 0.01)	0.34 (0.38)	0.71 (0.21)	96.10 (23.36)%
Planned RT	Null model	0.00 (0.00)	0.79 (0.21)	100.00 (0.00)%
	Time MLR	0.32 (0.35)	0.75 (0.20)	96.12 (16.54)%
	Speech MLR	**0.57 (0.32)**	**0.60 (0.18)**	**78.84 (25.42)%**
	Speech SVR (*C* = 1)	**0.58 (0.32)**	**0.60 (0.18)**	**78.75 (25.05)%**
	Speech SVR (*C* = 0.01)	0.51 (0.32)	0.66 (0.18)	87.49 (24.52)%
Memory	Null model	0.00 (0.00)	0.81 (0.19)	100.00 (0.00)%
	Time MLR	0.08 (0.38)	0.84 (0.19)	103.30 (9.17)%
	Speech MLR	**0.49 (0.31)**	**0.66 (0.19)**	**84.16 (26.87)%**
	Speech SVR (*C* = 1)	**0.49 (0.32)**	**0.67 (0.19)**	**84.28 (26.55)%**
	Speech SVR (*C* = 0.01)	0.36 (0.35)	0.76 (0.18)	96.31 (25.23)%
Cognition	Null model	0.00 (0.00)	0.77 (0.22)	100.00 (0.00)%
	Time MLR	0.16 (0.39)	0.78 (0.21)	102.26 (12.45)%
	Speech MLR	**0.43 (0.34)**	**0.69 (0.22)**	**93.94 (30.96)%**
	Speech SVR (*C* = 1)	**0.44 (0.34)**	**0.69 (0.22)**	**93.41 (30.35)%**
	Speech SVR (*C* = 0.01)	0.39 (0.34)	0.72 (0.21)	97.12 (27.42)%

*Average metrics are displayed with SDs shown in parentheses. Bold shows best performing systems*.

Table 4 shows that predictions based on time features alone are similar to those of the null model, which just allocates a *z*-score of 0 to all predictions. The time MLR showed slightly better performance on the planned RT test than on the other tests, with a correlation of 0.32. This is unsurprising since the analysis in the Section “Analysis of Psychophysiological Tests” showed that the planned RT test score was the only one significantly affected by sleep latency. For the predictions based on speech features, the error varied between 79 and 97% of the null model, with the planned RT being best predicted, the simple RT and memory tests being predicted less well, and the cognition test predicted poorest. The MLR and SVR (*C* = 1) models performed very similarly. Given that the MLR and SVR techniques are quite different in their model building approach, this gives confidence that the cross-validation procedure was fair. The SVR (*C* = 0.01) model performed worse that the SVR (*C* = 1) model, perhaps indicating that the *C* = 0.01 results were underfitted.

**Figure 3:**
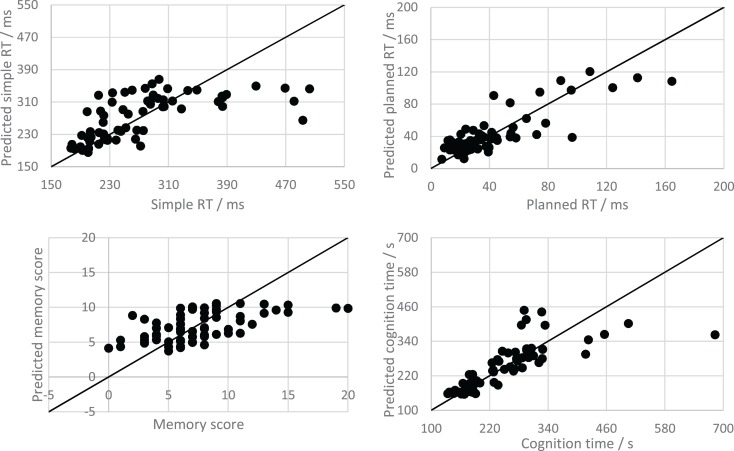
**Scatter plots showing the relationship between “time only” model predictions and observations for the psychophysiological tests**. The solid line is the line *y* = *x*, which shows all possible perfect predictions.

Although some indication of the model performance can be gained by observing the correlation coefficients and RAEs of the test *z*-scores, it is easier to interpret when the predictions and observations are de-normalized so that error can be considered in its original units. Figure 3 shows scatterplots indicating the relationship between the PPT scores and the de-normalized predictions for all test folds of a single 10-fold cross-validation of the time-features only MLR model (for each PPT). Recalculating the correlations and MAEs for the denormalised predictions of the time based MLR model gives *R* = 0.64, 0.83, 0.56, and 0.78, and MAE = 41.90 ms, 12.42 ms, 2.54 errors, and 35.55 s, for the simple RT, planned RT, memory, and cognition tests, respectively. The correlations are much higher than those calculated based on the *z*-standardized data, indicating that the subject means are responsible for the largest portion of the prediction accuracy (i.e., individual differences in subject performance account for a large part of the variation in the data).

**Figure 4:**
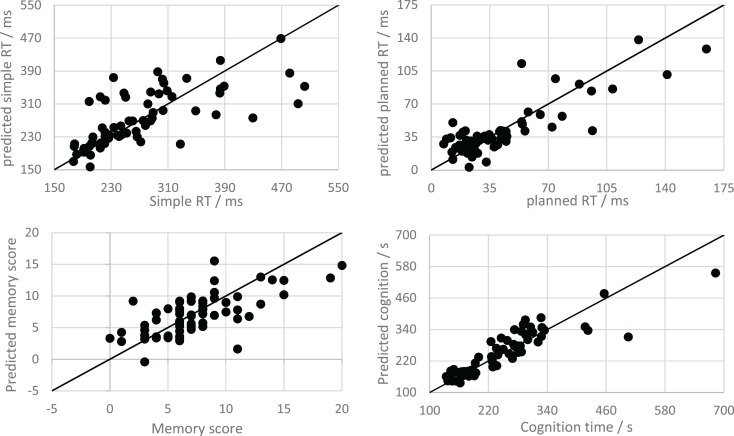
**Scatter plots showing the relationship between speech MLR model predictions and observations for the psychophysiological tests**. The solid line is the line *y* = *x*, which shows all possible perfect predictions.

Figure 4 shows the predictions for all test folds of a single 10-fold cross-validation of the speech MLR model (for each PPT). Recalculating the correlations and MAEs for the denormalised predictions of the speech-based MLR model gives *R* = 0.69, 0.84, 0.67, and 0.90, and MAE = 38.62 ms, 11.56 ms, 2.25 errors, and 27.43 s, for the simple RT, planned RT, memory, and cognition tests, respectively. Both correlations and MAEs are improved upon the time based models in every case. Dividing the MAEs by the full range of scores for each test gives error proportions of 11.9, 7.36, 11.26, and 5.00%. This indicates that all trained models were capable of making reasonably accurate predictions.

In summary, the speech-based models showed an improvement over the time based models in their prediction of PPT scores and had average prediction errors ranging from 5 to 12% of their range, and correlations ranging from *R* = 0.67 to 0.90.

## Summary and Conclusion

A sleep deprivation study was carried out during which trained aeronautical professionals performed a series of PPT and made a series of recordings of read speech. Analysis of the test scores showed a variation over time that was only weakly correlated with either sleep latency or phase. This unexpected result may have been due to the ability of the subjects to recruit additional mental effort to perform well at times unrelated to the duration of sleep deprivation or time of day. That is, the observed variation in test scores may have had more to do with variation in levels of motivation than simply time spent awake.

Analysis of the speech recordings showed that measurable and systematic voice changes occurred over the duration of the experiment. Some voice features seem to change over the duration of sleep deprivation and may be used to make predictions of sleep latency with an error of about 10 h on average. The change in voice features over the circadian cycle was much weaker, however, and could not be shown to be significantly different from a null model. Assuming that 60 h sleep deprivation represents maximum fatigue, this suggests that speech may be used to estimate fatigue level to approximately one part in six.

Significantly, voice features were also able to improve the predictions of test scores made within about 3 h of the recording over those that could be obtained by time alone. The prediction error of the best speech models were 10–18% smaller than time alone model. On average, over the four test types, scores could be predicted to within about 5–12% of their typical range from the speech recordings. The most improved test score prediction performance was obtained for the planned RT test, which was also the test score most strongly correlated with sleep latency. The other test scores were not only well correlated with sleep latency but also showed improved prediction by the speech models. This may be explained by the fact that different speech models were built for each PPT type, and that these may have tapped into different speech features.

That PPT scores are better predicted from the speech features than from time may be due to some common cognitive or physiological basis for test performance and speech performance. The ability of an individual to recruit additional mental effort despite lack of sleep on some occasion may have affected both test scores and voice features, and it is worth noting the possibility that the aeronautical professionals taking part in this study might have had a special aptitude for performing well while sleep deprived. If so, this would limit the generalizability of the specific models trained here, but it seems likely that new models could be trained on different subjects with similar performance.

A significant limitation of this study is the relatively small corpus size, which casts doubt on the generalizability of the models to new data and subjects. In addition, it was not possible to consider the effects of speaker gender, language, or accent, and train separate models where applicable. In a practical implementation such considerations might allow for improved prediction performance, however, the prediction performance obtained in the current work does demonstrate the validity of the model building approach, and makes explicit the tractability of the problem. The fact that both MLR and SVR models produce good predictions is evidence that the evaluation by cross-validation provides a fair estimate of likely performance on new data. If the models only worked by fortuitous selection of training vectors, we would see much weaker performance in the MLR model where a parametric model of feature values is constructed.

Additionally, it is important to note that the use of gaussianized speech features in the model construction procedure has important practical implications. In any implementation of this approach for the prediction of fatigue for a new speaker, it will be necessary to establish an enrollment stage in which the voice characteristics of the speaker are established before any prediction can be made.

The main finding of this work was that objective and continuous measures of fatigue can be made from analysis of the voice. The predictive models of fatigue demonstrate the tractability of the problem and the viability of this machine learning approach.

## Conflict of Interest Statement

The authors declare that the research was conducted in the absence of any commercial or financial relationships that could be construed as a potential conflict of interest.
